# Environmental and ecological factors influencing the spillover of the non-native kelp, *Undaria pinnatifida*, from marinas into natural rocky reef communities

**DOI:** 10.1007/s10530-017-1610-2

**Published:** 2017-11-10

**Authors:** Graham Epstein, Dan A. Smale

**Affiliations:** 1 Marine Biological Association of the United Kingdom, The Laboratory, Citadel Hill, Plymouth, PL1 2PB UK; 2 Ocean and Earth Science, National Oceanography Centre Southampton, University of Southampton, Waterfront Campus, European Way, Southampton, SO14 3ZH UK

**Keywords:** Invasive, Alien species, Wakame, Marina, Distribution, Management

## Abstract

The non-native kelp, *Undaria pinnatifida*, is considered one of the world’s worst invasive species. The northeast Atlantic is a hotspot of *Undaria* invasion, yet there is limited knowledge on its invasion dynamics. In the UK its distribution is strongly associated with artificial structures, primarily marina and harbour pontoons, with relatively few records of *Undaria* on natural substrates. Here, the southwest UK is used as a case region, to explicitly link *Undaria* distribution-abundance patterns in artificial marina habitats with those in natural rocky reef habitats. Using a mixture of in situ recording and video survey techniques, *Undaria* was found at all thirteen marina sites surveyed; but in only 17 of 35 rocky reef sites, all of which were in 2 of the 5 larger systems surveyed (Plymouth Sound and Torbay). The distribution-abundance patterns of *Undaria* at reef sites were analysed using zero-inflated models. The probability of finding *Undaria* on rocky reef increased with increasing proximity to marinas with high abundances of *Undaria*. Total propagule pressure from marinas also increased the probability of occurrence, and was positively related to *Undaria* abundance and cover at reef sites. Increases in the cover of native kelps, *Laminaria* spp., and wave exposure at reef sites were linked to a reduced probability of *Undaria* occurrence, and lower abundance and cover. Identifying high risk areas, natural boundaries and factors affecting the spread and abundance of non-native species in natural habitats is key to future management prioritisation. Where *Undaria* is confined to artificial substrates management may be deemed a low priority. However, the results of this study suggest that controlling the abundance and propagule pressure in artificial habitats may limit, to some extent, the spillover of *Undaria* into natural rocky reef habitats, where it has the potential to interact with and influence native communities.

## Introduction

Artificial structures are strongly associated with the colonisation of marine non-native species (NNS) (Bulleri and Chapman 2010; Glasby et al. 2007; Ruiz et al. 2009). Seawalls, pontoons, buoys and aquaculture equipment are generally found in more nutrient enriched, low salinity, sediment loaded or polluted environments, as a result of being located in areas of intensified human activity. This distinct physical and biological environment provides a habitat to which many native species are not adapted and can therefore harbour a distinct assemblage (Bulleri and Chapman 2010; Glasby et al. 2007; Ruiz et al. 2009). These environments also coincide with major introduction pathways and therefore often support a high propagule pressure of NNS (Bax et al. 2003).

Recreational boating is now recognised as one of the major vectors and introduction pathways of NNS, which may be transported via hull fouling or within ballast and bilge water (Airoldi et al. 2015; Clarke Murray et al. 2011; Fletcher et al. 2017; Zabin 2014). Floating pontoons within harbours and marinas have therefore been identified as key habitats for NNS and are now the focus of numerous monitoring and assessment programs (e.g. Arenas et al. 2006; Bishop et al. 2015; Connell 2001; Foster et al. 2016; Glasby et al. 2007). This has led to many new records of NNS originating from marina habitats over the last two decades (e.g. Arenas et al. 2006; Fletcher and Manfredi 1995; Ryland et al. 2014). Although this may be because of increased sampling effort, the abundance and richness of NNS is also considerably higher within marinas when compared to adjacent natural hard-bottom habitats (Airoldi et al. 2015; Connell 2001; Dafforn et al. 2012; Glasby et al. 2007). This would suggest that marinas act as ‘strongholds’ for NNS.

Species which were initially recorded in marinas can now be found in a variety of natural habitats, albeit normally at lower abundances (e.g. Connell 2001; Dafforn et al. 2012; Farrell and Fletcher 2006; Minchin and Duggan 1988; Ryland et al. 2009). The interconnected nature of the marine environment makes it hard to definitively link the spread of species from artificial structures to natural coastal habitats. However, as marinas generally comprise large areas of artificial substrate with high abundances of NNS, they can facilitate the development of substantial propagule pressure (Arenas et al. 2006; Foster et al. 2016; Glasby et al. 2007). The proximity of many of these marinas to natural hard-bottom substrates means the ‘spillover’ of NNS from marinas to nearby natural habitats is highly likely in many systems.

The ability to separate NNS with negligible ecological impacts from those that pose significant risk to native communities is critically important for biodiversity conservation and effective management of natural resources (Blackburn et al. 2014; Jeschke et al. 2014). This is because of the need to prioritise management and control of the large number of marine NNS already established globally (Bax et al. 2003; Minchin et al. 2013; Molnar et al. 2008). The abundance and range of NNS are generally considered as key aspects of impact assessments (Parker et al. 1999; Thomsen et al. 2011). However, due to their ‘conservation value’, the ecological impact of NNS in natural habitats is generally considered as greater to that of NNS on artificial structures (Kueffer and Daehler 2009). Although many other factors will influence the overall effect an NNS has on native communities, understanding processes driving the abundances, distributions and rates of transfer of NNS within natural habitats is paramount.

There are thought to be approximately 350 species of non-native marine macroalgae worldwide and at least 17 in the UK, accounting for 20–30% of all marine NNS (Minchin et al. 2013; Schaffelke et al. 2006; Thomsen et al. 2016). Marine macroalgae can function as ecosystem engineers with the potential to cause significant economic and ecological impacts (Schaffelke and Hewitt 2007; Thomsen et al. 2009; Williams and Smith 2007). The cold-temperate kelp *Undaria pinnatifida* is one of only two marine macroalgae (along with *Caulerpa taxifolia*) included in the Invasive Species Specialist Group list of the 100 most invasive species of the world (Lowe et al. 2000). *Undaria pinnatifida* (hereafter referred to as *Undaria*) is native to the northwest Pacific, where it inhabits rocky coastlines of Japan, Korea, Russia and China (Koh and Shin 1990; Saito 1975; Skriptsova et al. 2004). It is also a major species for seaweed mariculture, and is predominantly grown using longline ropes (Peteiro et al. 2016; Yamanaka and Akiyama 1993). As a NNS *Undaria* can now be found in many parts of the northeast and southwest Atlantic, southwest and east Pacific, and the Tasman Sea (James et al. 2015).

The impact of *Undaria* on recipient communities is thought to be highly variable and site-specific. Current evidence indicates that in the majority of cases *Undaria* seems to act as a passenger of ecosystem-change, requiring a level of disturbance or high resource availability in order to establish and proliferate, while having minimal impact on native communities (Forrest and Taylor 2002; South et al. 2015; South and Thomsen 2016; Valentine and Johnson 2005). However, there is evidence that in some settings *Undaria* may impact macroalgal, invertebrate and fish communities (Carnell and Keough 2014; Casas et al. 2004; Farrell 2003; Irigoyen et al. 2010, 2011). More research is needed to better understand the range of impacts *Undaria* may have on recipient communities; there is a clear need for long-term manipulative studies that incorporate a range of responses at the individual, population and community level.

The initial introduction of *Undaria* outside of its native range was via accidental import with shellfish into French Mediterranean coastlines in 1971 (Floc’h et al. 1991; Perez et al. 1981), followed by intentional introductions for cultivation into Brittany in 1981 (Perez et al. 1981). Accidental or intentional Introductions for farming were initially the primary vector of transport in the northeast Atlantic (Peteiro et al. 2016; Voisin et al. 2005). However, over time and across other regions, long distance dispersal of *Undaria* was predominantly thought to be via fouling on the hulls of commercial vessels (Forrest et al. 2000; Hay 1990; Silva et al. 2002; Voisin et al. 2005). Within certain regions, *Undaria* is strongly associated with aquaculture infrastructure and secondary spread is thought to have occurred between aquaculture sites (James and Shears 2016). In the north east Atlantic secondary spread and range expansions are thought to have been facilitated by fouling on recreational vessels and transport to nearby ports and marinas (Fletcher and Farrell 1999; Kaplanis et al. 2016; Veiga et al. 2014; Zabin 2014).

In its non-native range, *Undaria* is characterised by its prevalence on artificial rather than natural substrates (Cremades et al. 2006; Fletcher and Farrell 1999; Floc’h et al. 1996; Kaplanis et al. 2016; Russell et al. 2008; Veiga et al. 2014). Many of the records of *Undaria* therefore originate from ports, marinas and aquaculture sites (Fletcher and Manfredi 1995; Hay and Luckens 1987; Kraan 2016; Meretta et al. 2012; Silva et al. 2002). Both marinas and aquaculture sites contain artificial substrates which are held at a constant shallow depth, providing ideal light conditions for the growth of *Undaria* (Cremades et al. 2006; Fletcher and Farrell 1999; Grulois et al. 2011; James and Shears 2016; Minchin and Nunn 2014). As a non-native, *Undaria* can also be found in a variety of natural habitats including seagrass beds and mixed sediment communities, although it is most commonly found on rocky reef (Dellatorre et al. 2014; Hewitt et al. 2005; Martin and Bastida 2008; Minchin and Nunn 2014; Russell et al. 2008). Due to its low natural dispersal ability, following introduction into a non-native region, the natural spread of *Undaria* can be relatively slow (Farrell and Fletcher 2006; Floc’h et al. 1991; Kaplanis et al. 2016). However, in many cases it has been suggested that the presence of *Undaria* in natural habitats is linked to source populations in nearby artificial habitats (Farrell and Fletcher 2006; Floc’h et al. 1996; Grulois et al. 2011; James and Shears 2016; Russell et al. 2008).

**Fig. 1:**
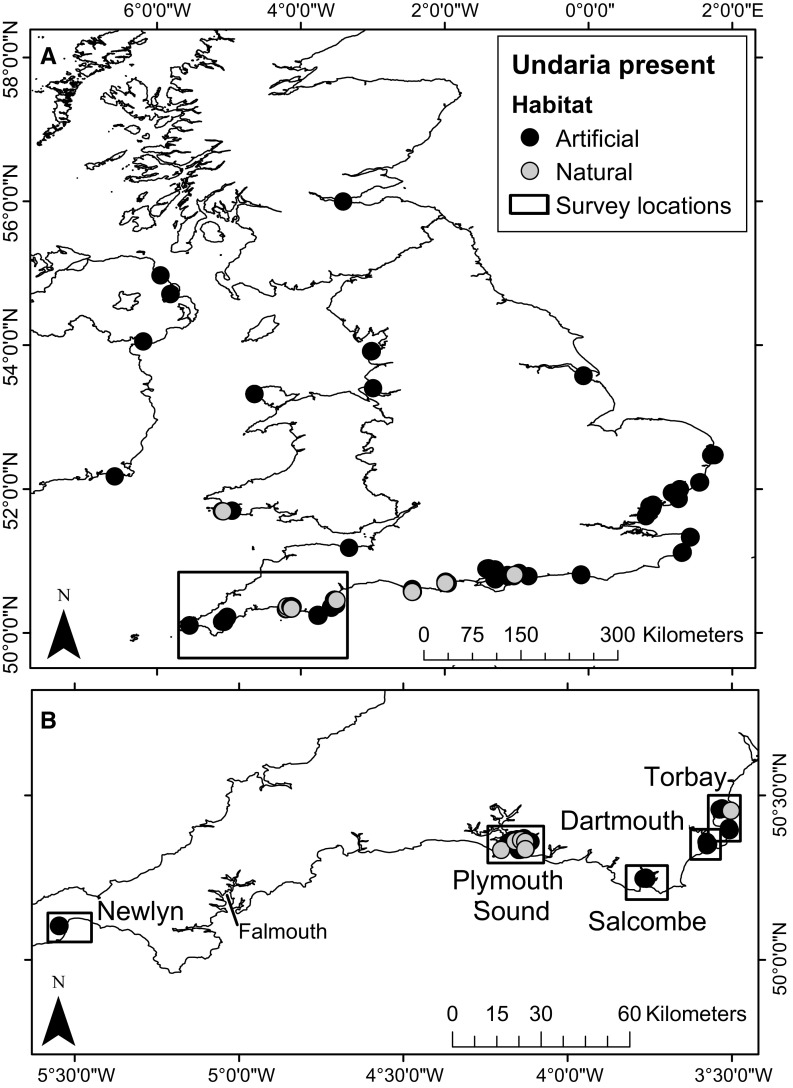
**a** Records of *Undaria* occurrence in the UK and Ireland (Kraan 2016; Minchin et al. 2017; NBN 2016). Black box indicates survey region. **b** Locations selected for survey in the southwest UK survey region (black boxes). *Undaria* records are present around Falmouth, although not shown here as this location was not part of the current study. Within both maps records from natural habitats (grey points) shown on top of those from artificial habitats (black points). Habitat type was determined based on survey and site information from the original records

In the UK *Undaria* was first recorded in 1994, attached to floating marina pontoons in Port Hamble (Fletcher and Manfredi 1995). By 1999, *Undaria* had spread to other marinas and harbours along the south coast of England (Farrell and Fletcher 2006). Currently, although the majority of records still originate from southern England, the species has been recorded on the south, east and west coasts of England, on the east coast of Northern Ireland and the Republic of Ireland, and in Scotland at Queensferry (Fig. 1). In the vast majority of locations these records are from artificial structures, primarily marina and harbour pontoons (Fletcher and Farrell 1999; Heiser et al. 2014; Kraan 2016; Minchin and Nunn 2014; NBN 2017). Despite its widespread distribution, *Undaria* has been recorded on natural substrates in relatively few areas of the UK (Farrell and Fletcher 2006; Fletcher and Farrell 1999; Heiser et al. 2014; NBN 2017). This may be because it is largely confined to artificial habitats, or it could be generally under-recorded in shallow natural habitats that are more difficult to sample.

Here, the southwest UK is used as a case region, to investigate links between *Undaria* distribution-abundance patterns in artificial habitats and those observed within natural rocky reef habitats. Attributes of both the marina and coastal sites are quantified to identify factors which may influence the distribution and abundance of *Undaria* on natural rocky reefs. The overall objectives of the study were: (1) to determine whether *Undaria* is largely confined to artificial habitats or whether it has spread to natural rocky reef; (2) to quantify ecological and environmental factors that may influence the spread of *Undaria* into natural habitats and explicitly link them with observed distribution-abundance patterns; and (3) to consider how these findings may influence the design of appropriate management responses.

## Methods

### Survey locations

Records of *Undaria* on the south coast of Devon and Cornwall were obtained from the National Biodiversity Network Gateway (NBN 2016). These were largely confined to marina environments, with relatively few records of *Undaria* from natural rocky reef habitats (see Farrell and Fletcher 2006; Heiser et al. 2014 for further details). Based on existing records five locations were chosen for survey; Torbay, Dartmouth, Salcombe, Plymouth Sound and Newlyn (Fig. 1). Of these, two are designated as protected areas due to their high conservation value; Plymouth Sound (Special Area of Conservation) and Salcombe Estuary (Special Site of Scientific Interest). All surveys were completed during summer (June–August) as this is the season when the main recruitment and growth periods of *Undaria* would be expected to have ended, and therefore the populations should be at a plateau; however it would also be the start of the main annual senesce (Arnold et al. 2016; Heiser et al. 2014; Minchin and Nunn 2014; Murphy et al. 2017). Temporal variation in recruitment, growth and senescence stages between locations may have had an influence on overall abundance, biomass or cover estimates. However, long-term sampling across all locations would be needed to remove any temporal influence, which falls outside the scope of the current study. The 2.5 month restricted summer survey period was therefore considered to be an appropriate design for this survey, with similar time periods being used for other studies of *Undaria* within the UK (Arnold et al. 2016; De Leij et al. 2017; Farrell 2003; Heiser et al. 2014; Minchin and Nunn 2014; Minchin et al. 2017).

### Marina surveys

Marinas containing records of *Undaria* in NBN (2016) were visited during 13th June–25th August 2016. The same observer walked the full extent of the marina pontoons and gave a single categorical score for *Undaria* percent cover of submerged floats using a SACFOR scale (Superabundant [S > 80%], Abundant [A 40–79%], Common [C 20–39%], Frequent [F 10–19%], Occasional [O 5–9%], Rare [R 1–5%], None [N 0%]). The areal extent of each marina was calculated using Google aerial imagery © 2016, and a measure of *Undaria* propagule pressure for each marina was calculated by multiplying the median percent cover value from the SACFOR category by aerial extent. Values were summed for marinas within a given location to give an estimate of total marina propagule pressure (hereafter referred to as ‘propagule pressure’). Although this is a relatively coarse proxy for propagule pressure (it was not feasible to collect more precise measures of spore density or recruitment density across such broad spatial scales), it is representative of the standing stock of mature sporophytes and no clear differences in the relative abundance of reproductively-active sporophytes were observed between marinas. Time of first record in each marina was also collected from NBN (2016).

The coverage of both native and non-native large brown macroalgae on marina pontoons can be highly spatially variable within a single marina. This is dependent on a variety of factors including aspect, shading, water depth, exposure, shielding from vessels and disturbance. Therefore, to get a comparative measure of abundance and biomass of *Undaria* at each marina, the area supporting the greatest coverage of brown macroalgae (assessed visually during the SACFOR search) was targeted for further high resolution sampling. This was typically the outermost pontoons nearest to the marina entrance, where there is little or no shielding from vessels, greater water depth and stronger water flow (Epstein pers obs). Within the selected area, 10 replicate 0.25 m^2^ quadrats where haphazardly placed against the side of submerged floats. All *Undaria* were removed from the quadrat, enumerated, and the total biomass quantified (g FW). Values were averaged over the ten quadrats at each marina to yield comparable values of abundance and biomass.

### Coastal surveys

Using ArcMap 10.3.1 the mean high water spring (MHWS) coastline of each location was divided into equal segments of 3.75 km in length. Those coastal segments closest to the marina sites identified above were selected first. A single survey site was haphazardly chosen within the segment based on shore access and presence of suitable rocky substrate—identified using Google aerial imagery © 2016 and by carrying out site visits. The first segment was generally seaward of each marina due to a lack of suitable rocky habitat on the estuary/river side of the artificial habitat. If a coastal segment contained no shore access, or there was a lack of suitable rocky substrate, it was removed from the study area and site selection continued to the adjacent segment. Survey sites closest to marinas were surveyed first, with each subsequent survey moving to the adjacent segment, therefore extending the range of the study area from the marina site. If two consecutive survey sites contained no *Undaria*, survey effort moved to the opposite side or shore from the marina. If 2 days of survey effort (3–4 sites) recorded no *Undaria* within a given location no further sites were sampled.

Surveys were completed by snorkel at low slack-tide during 2nd July–30th August 2016. In order to maintain a similar tidal height on the substrate, large spring tides were avoided, leading to tidal heights of between 0.7 and 2.0 m above chart datum at the time of survey. At each site, four 25 m transects were laid using a weighted line, each separated by approximately 25 m. Transects were placed haphazardly, but were stratified to areas of suitable rocky substrate within the intertidal/subtidal fringe zone, which was covered by ~ 0.5–1.5 m of water at the time of survey. Video of the macroalgal canopy along the transect was collected using a Panasonic Lumix FT5 waterproof camera fitted to an underwater tray and handle. A 65 cm scale was fixed to the front of the camera tray in order to maintain the video at an approximate set distance above the canopy, to standardise the area of observation (approximately 20 m^2^ per transect). Both horizontal and vertical substrates were included in the video, dependent on the topography at a given site. For each transect the substrate was categorised by the percent contribution of bedrock, boulders (> 500 mm), cobbles (60–500 mm), gravel (5–59 mm) and sand (< 5 mm), which was estimated by eye. This was converted to a univariate measure of substrate stability using the formula:

$$Substrate\,stability = \% Bedrock + \frac{2\,*\,\% Boulders}{3} + \frac{\% Cobbles}{3}$$

**Fig. 2:**
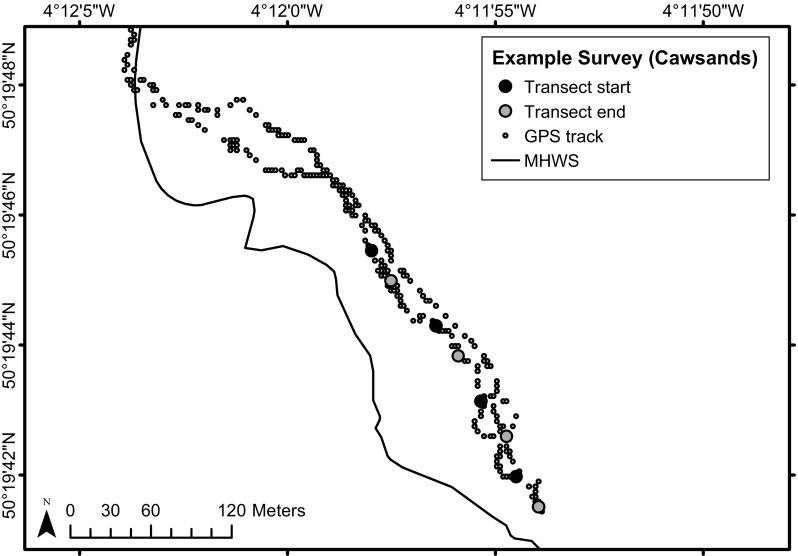
Example layout of survey at a coastal reef site. The geographic position of each transect (start—black, end—grey) was estimated by matching the time at the start and end of the video to a GPS track (small grey dots) recorded from a hand-held GPS, housed in a swim buoy attached to the surveyor

The percent canopy cover of *Undaria*, measured on a SACFOR scale, was also recorded in situ for each transect; this visual census incorporated macroalgal canopies ~ 2 m each side of the transect line and therefore covered a greater area than the video transects. The geographic position of each transect was estimated by matching the time at the start and end of the video to a GPS track recorded from a Garmin etrex GPS, housed in a swim buoy attached to the surveyor (Fig. 2).

Following the survey, each video transect was viewed twice. On the first view the video was played frame-by-frame and the number of *Undaria* (both entire and partial plants) were counted. If *Undaria* was found during the in situ search, but was not counted within the video transect, a nominal value of 1 was given, to distinguish these transects from true absences. This resulted in two measures of *Undaria* for each transect: (1) the in situ SACFOR measure at wider spatial scale but coarser resolution and (2) the abundance measure at smaller spatial scales but finer resolution obtained from the video transects. On the second viewing of the video transects the percent canopy cover of other large brown canopy-forming macroalgae (*Laminaria* spp., *Saccorhiza polyschides*, *Saccharina latissima*, *Himanthalia elongata*, *Chorda filum*, *Sargassum muticum* and *Alaria esculenta*) was estimated on the SACFOR scale.

### Other coastal site characteristics

The dispersal distance between each coastal survey site and the nearest marina was calculated in ArcMap 10.3.1. The primary method of natural dispersal of *Undaria* is from spores, which have low natural dispersal abilities (Forrest et al. 2000; Saito 1975; Schiel and Thompson 2012). Long-distance drift of mature sporophytes is considered a potential secondary method of natural dispersion (Forrest et al. 2000; Grulois et al. 2011), which may create distinct dispersal distances. However, along-shore distance was considered the most appropriate measure of dispersal distance from marina to reef site, due to the low buoyancy of mature sporophytes, the predominance of spore mediated natural dispersion, and the likely correlation between along-shore distance and linear distance to marinas. Polylines were creating running from the centre of each study site along the MHWS shoreline to the nearest marina. Estuarine channels of less than 500 m in width were not considered as geographical barriers to *Undaria* dispersal (Forrest et al. 2000; Russell et al. 2008), and therefore a straight line was drawn across these points. Human mediated dispersal is highly stochastic, with both long and short distance vectors. This has the potential to influence connectivity between sites, however due to its high variation, estimating a true value is highly challenging, while calculating a proxy such as quantification of vessel movements in each location was unfeasible and falls outside the scope of the current study. The maximum abundance and biomass of *Undaria* at the nearest marina was also applied as a coastal site characteristic.

Local wave exposure was calculated by manually summing the distance to land from the centre of each study site for each of 18 radial points separated by 20°. The maximum radial distance was set as 200 km as this is approximately where the fetch is considered large enough for wave conditions to be fully developed for UK coastal locations (Burrows et al. 2008). Distance at each radial point was calculated using a high resolution polyline of UK MHWS and will therefore be strongly influenced by small, site level topography and barriers. A lower resolution, “segment-level”, measure of exposure was calculated from the Burrows et al. (2008) UK fetch model. Within this model the UK coastline is divided into 200 m scale grid cells and wave fetch is determined as the distance to the nearest land cell in 16 radial sectors, based on three resolutions of searches of the surrounding cells, up to a distance of 200 km (Burrows et al. 2008). For each coastal cell the mean values of fetch with its two immediately adjacent cells is then calculated to create a measure of exposure (Burrows et al. 2008). For the closest coastal cell to the centre of each study site, this final exposure value was used as a measurement of segment exposure.

### Statistical analysis

Zero-skewed distribution and abundance data is frequently found in studies on rare or restricted-range species (Martin et al. 2005). During this study, *Undaria* was absent in 57% of transects. Analysis was therefore carried out using zero-inflated models as the preponderance of zeros would cause high overdispersion within ordinary count models (Poisson and negative binomial). Zero-inflated models have commonly been used to identify environmental and ecological factors influencing the distribution-abundance patterns of rare or restricted-range aquatic species, including marine NNS (e.g. Anton et al. 2014; Cambie et al. 2017; Erhardt and Tiffan 2016; Fletcher et al. 2013; Hoogenboom et al. 2015). Factors affecting the distribution and abundance of *Undaria* at coastal sites were assessed using zero-inflated negative binomial models (ZINB, Zuur et al. 2009). A ZINB is a mixture model whereby zero values are modelled as coming from two parts. In the first instance, a binomial GLM models the probability of measuring a zero based on selected covariates and presence-absence of the response—hereafter referred to as the ‘zero model’ or *π*_*i*_. The second part models remaining variation in zeros, and positive values with a negative binomial GLM—hereafter referred to as the ‘count model’ or *µ*_*i*_ (Zuur et al. 2009). All models were run in R 3.2.2 (R Core Team 2015) using the *zeroinfl* function from the *pscl* package (Zeileis et al. 2008).

The two *Undaria* response variables, abundance counts from the video transects and SACFOR cover from in situ surveys (median value from the SACFOR category rounded to the nearest percent), were modelled separately. Predictor variables used to model the response included both ecological and environmental attributes of each site. Specifically, ecological descriptors were the percent cover of *Laminaria* spp., *S. latissima* and *S. polyschides* on natural reef; the abundance of *Undaria* at the nearest marina, and local marina propagule pressure; whereas substrate stability, distance to nearest marina and wave exposure described the environment. Percent cover of *Laminaria* spp., *S. latissima* and *S. polyschides* were calculated as the median value from the SACFOR category (expressed as a decimal value). On average these three species accounted for 91% of all brown macroalgal canopy (excluding *Undaria*), and were therefore considered to characterise the associated community as a whole. During the marina surveys it was noted that the annual senescence of the *Undaria* lamina had progressed at different rates at each marina. This could greatly influence the overall biomass and therefore ‘biomass at nearest marina’ was not used as a predictor variable. The holdfasts and stipes of plants generally stay attached to the substrate for some time following senescence of the blade, and therefore, abundance at nearest marina was considered a reliable descriptor. Time since first record in each location was not used as a predictor variable because the metric is (1) highly influenced by historic survey effort which is unequal between locations; (2) unlikely to reflect the actual date of introduction due to lack of absence records in many cases; and (3) likely to be highly related to the abundance at and propagule pressure from marinas.

Collinearity in predictor variables was tested using Pearson correlation coefficients and variance inflation factors (VIF) using the *pairs* and *corvif* functions (Zuur et al. 2009). The need to transform variables before testing for collinearity was assessed graphically. No transformations were needed, and no collinearity was identified (r ≤ 0.6, VIF < 2.6, for all variables).

Models were fitted using backward selection. Initially a full ZINB with all predictor variables included within both the zero and count models was constructed. The coefficient with the lowest significance value was dropped, and the model rerun. This was repeated until all coefficients within the model had a *p* value < 0.01. Each model was compared to the subsequent nested model using a likelihood ratio test using the *lrtest* function within the *lmtest* package (Zeileis and Hothorn 2002). Second-order Akaikes information criterion (AICc) were calculated for all models using the *AICc* function in the *AICcmodavg* package (Mazerolle 2016). Optimal models were selected based on both likelihood ratio tests and AICc values, however AICc values with a difference of less than 2 were not considered significant. The selection of a ZINB over a zero inflated Poisson model (ZIP; where remaining zeros and positive values are modelled with a Poisson distribution) was due to over-dispersion in the count portion of the data. This was justified using a likelihood ratio test at both full and optimal models stages.

Model validation was carried out using diagnostic plots. Pearson residuals were plotted against fitted values from the optimal ZINB model, and against each explanatory variable. Observed values of the response were plotted against fitted values from the optimal ZINB, and model fit was tested using a simple linear regression (Pineiro et al. 2008). Relative importance of each coefficient was calculated as the percentage value of the z-statistic from the total absolute z value for each portion of the optimal models. To further examine the relationship between predictor and response variables binomial models were constructed for *Undaria* presence-absence and each predictor variable selected in the optimal zero model; while negative binomial models were constructed for each predictor selected in the optimal count models and positive abundance and SACFOR data. This was carried out using the *glm* function from base R (R Core Team 2015) and the *nb.glm* function from the *MASS* package (Venables and Ripley 2002).

Mapping was carried out within ArcMap 10.3.1. The *dplyr* package (Wickham and Francois 2015) was used for data manipulation and all graphs were created using *ggplot2* (Wickham 2009).

## Results

### Marina surveys

**Fig. 3:**
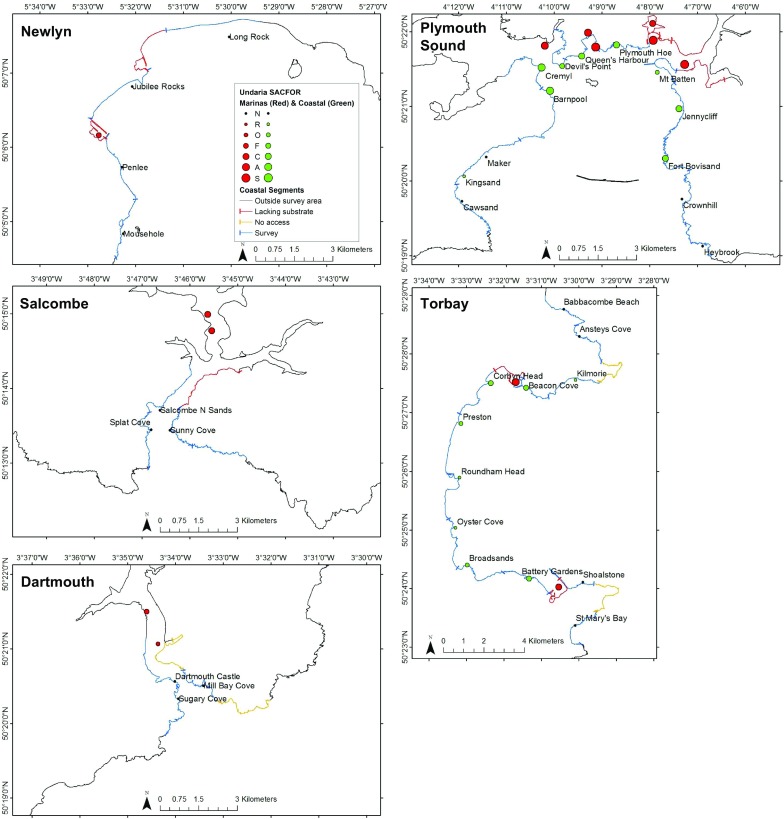
Undaria SACFOR at marina (red) and coastal (green) survey sites. Labels indicate the names of coastal survey sites. Where applicable *Undaria* absence is indicated by a black point. Ports which did not contain floating pontoons (such as north of Jubilee rocks, Newlyn) were not surveyed. Coastal segments are coloured to indicate where a survey was completed (blue), where no shore access was available (orange) and where natural rocky substrate was lacking and therefore no survey was carried out (red). Coastline which was outside of the scope of this survey is shown in black

*Undaria* was found at all thirteen marina sites surveyed (Fig. 3). The highest percent cover was in Plymouth Sound, with *Undaria* scored as Superabundant within three marinas. It was also the location supporting the highest abundance (50.9 ± 7.9 per 0.25 m^2^; mean ± SE) and biomass (2906.5 ± 413.6 g per 0.25 m^2^) of *Undaria* within a marina. The lowest percent cover within a single marina was at Dartmouth where *Undaria* was scored as Occasional. The lowest abundance (2.8 ± 1.0 per 0.25 m^2^) and biomass (270.4 ± 68.4 g per 0.25 m^2^), was recorded at marinas in Dartmouth and Newlyn respectively.

**Fig. 4:**
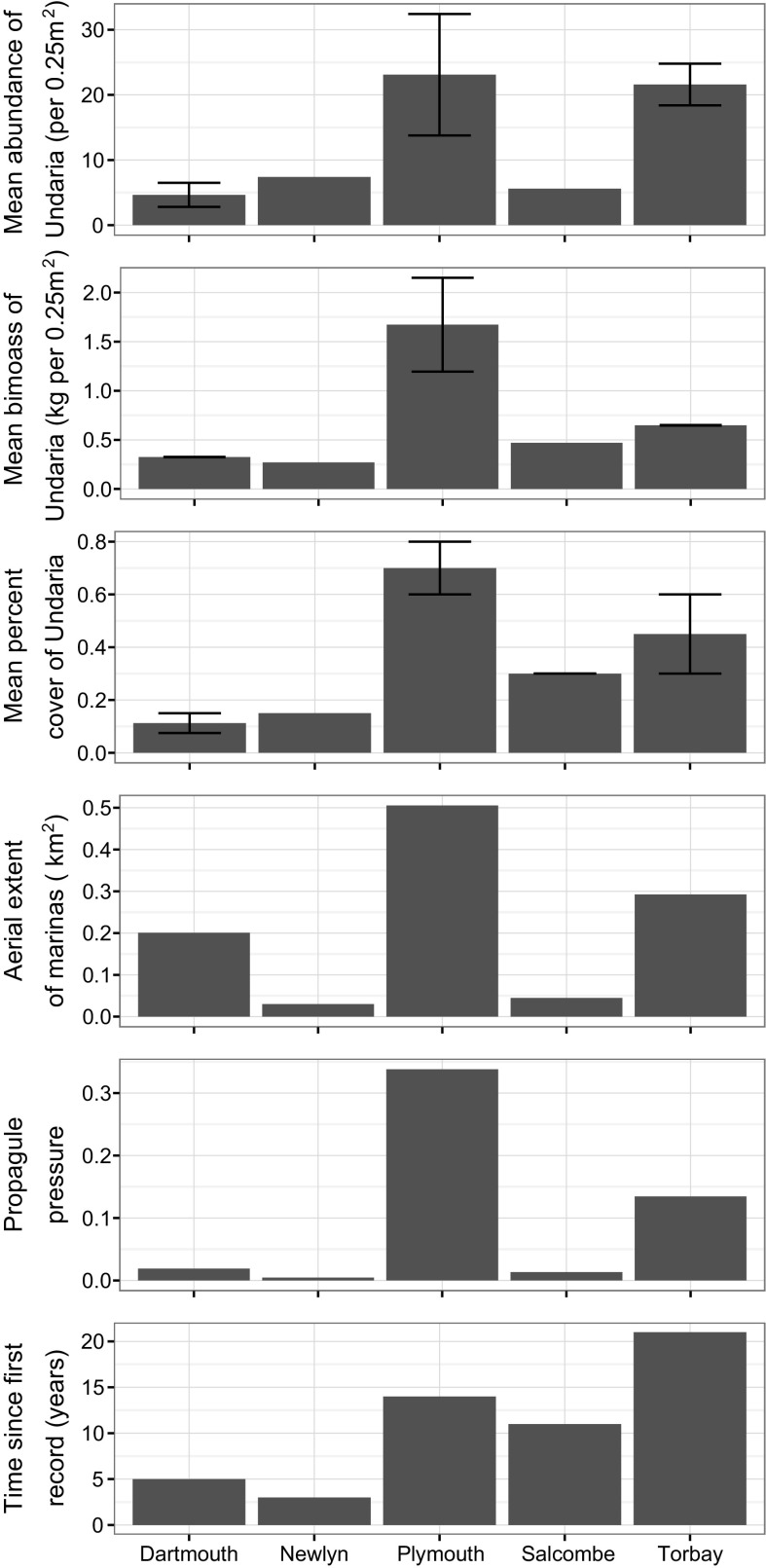
Attributes of surveyed marinas in each location. Abundance (inds. per 0.25 m^2^), biomass (kg per 0.25 m^2^) and percent cover (%) of *Undaria* calculated as a mean (± SE) of all surveyed marinas within a location (Salcombe and Newlyn only one marina surveyed). Areal extent of marinas (km^2^) and propagule pressure are a sum of all marinas, while time since first record is the earliest record for any marina within a given location

Overall, Plymouth Sound marinas had the highest mean abundance and biomass of *Undaria*. It was also the location with the largest mean percent cover (calculated from the median values from the SACFOR scales), total aerial extent of marinas and prologue pressure (Fig. 4). Torbay was the location with the earliest record of *Undaria* (1996), and had the second highest value for all factors (mean abundance, mean biomass, mean percent cover, total aerial extent of marinas and propagule pressure). Summary statistics for all locations are shown in Fig. 4.

### Coastal surveys

Across all locations a total of 35 coastal sites were surveyed (13 sites in Plymouth, 12 in Torbay, 4 in Newlyn, 3 in Dartmouth and 3 in Salcombe). *Undaria* was found at only 17 sites and within 60 of 140 transects, all of which were in Plymouth Sound and Torbay (Fig. 3). *Undaria* was not recorded at any coastal sites within Newlyn, Dartmouth or Salcombe (Fig. 3). The range of site characteristics found in each location is shown in Table 1.

**Table 1:** Range of site characteristics across each location

Location	Distance to nearest marina (km)		Site exposure (km)		Segment exposure (km)		Substrate stability (%)	
	Min	Max	Min	Max	Min	Max	Min	Max
Dartmouth	2.87	3.91	206.2	604.4	224.4	350.4	57	97
Newlyn	1.93	9.16	482.9	893.4	866.8	986.4	33	90
Plymouth	0.72	12.99	31.4	926.6	247.2	866.0	30	95
Salcombe	4.55	6.60	412.9	611.3	260.4	599.6	50	88
Torbay	0.99	13.09	38.7	803.2	490.0	1279.2	37	97

**Fig. 5:**
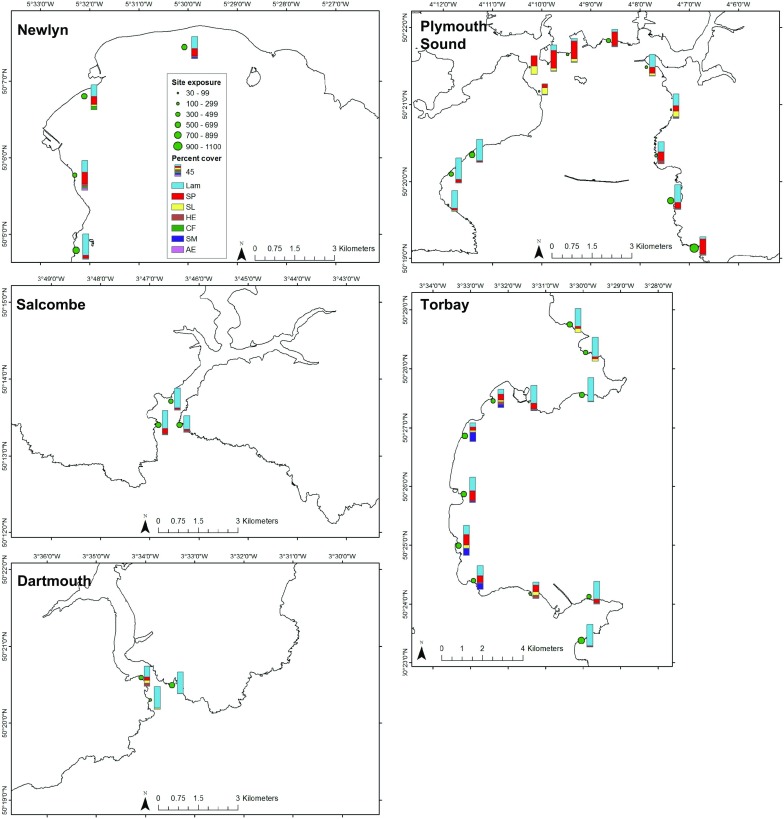
Site exposure (km) of each coastal survey site indicated by size of green point. The associated canopy community is shown to the right of each point as a stacked bar chart. Lam = *Laminaria* spp., SP = *Saccorhiza polyschides*, SL = *Saccharina latissima*, HE = *Himanthalia elongata*, CF = *Chorda filum*, SM = *Sargassum muticum*, AE = *Alaria esculenta*. Height of the bar is relative to percent cover of each species based on the SACFOR data

The structure of the associated brown macroalgal canopy varied considerably between sites, ranging from being entirely dominated by *Laminaria* spp. to comprising a far more mixed canopy of *Undaria*, *S. polyschides* and *S. latissima*. The average percent cover of each canopy-forming macroalgae at each site was calculated from the median values from the SACFOR categories at each transect and is shown in Fig. 3 (*Undaria*) and Fig. 5 (other canopy formers).

**Fig. 6:**
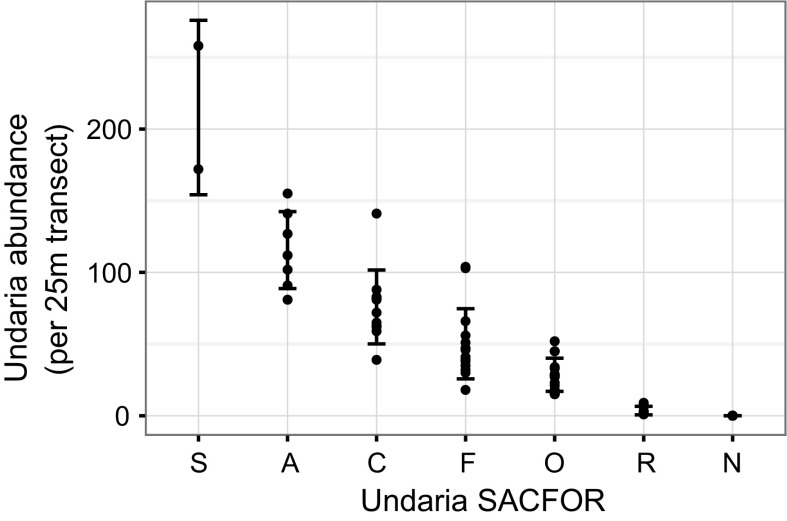
Relationship between *Undaria* SACFOR and abundance measured at each survey transect. Points indicate raw data at each transect. Bars indicate the mean abundance ± SD for each *SACFOR* category

The abundance of *Undaria* (counted within the video transects) was highly correlated to the in situ measure of *Undaria* percent cover (r = 0.93, calculated using the median values from the SACFOR category), however there was clear overlap in abundance values between different SACFOR categories (Fig. 6). *Undaria* was recorded as Superabundant within two transects at Barnpool (Plymouth Sound), where the maximum abundance was also recorded (258 within a single video transect) and was recorded as Rare within ten transects across seven sites in Torbay and Plymouth.

### Factors affecting the abundance and distribution on coastal reef

Using the backwards selection process eleven ZINB models were constructed for both the *Undaria* abundance and SACFOR response variables (Table 2). The lowest AICc value for *Undaria* abundance was 620.6 (A6), however five different models had a ∆AICc of less than 2 (A4 to A8) and were therefore considered for optimal model selection. Likelihood ratio tests indicated that a significant term was not dropped in the backwards selection until A8, although its significance was negligible (pSP from *π*_*i*_; χ^2^ = 3.8517, *df* = 1, *p* = 0.050). A8 was therefore chosen as the optimal model (Table 3). For the SACFOR response variable the lowest AICc was 496.2 (S6), with 4 models ∆AICc less than 2 (S5 to S8). Likelihood ratio tests indicated that a significant term was not dropped from the model until S9 (Site exp from *πi*; χ^2^ = 5.4353, *df* = 1, *p* = 0.020) and therefore S8 was chosen to be the optimal model. All coefficients in both optimal models were statistically significant with *p* values < 0.025 (Table 3).

**Table 2:** ZINB backwards selection process

Model	Dropped term	df	AICc	∆ AICc	Likelihood ratio test
	*Undaria abundance*				
A0	None	21	631.0	10.4	
A1	Dist marina from *µi*	20	628.3	7.7	χ^2^ = 0.0012 (*df* = 1, *p* = 0.972)
A2	Abund marina from *µi*	19	626.2	5.6	χ^2^ = 0.7155 (*df* = 1, *p* = 0.398)
A3	Stability from *πi*	18	624.4	3.8	χ^2^ = 0.8392 (*df* = 1, *p* = 0.360)
A4	Seg exp from *πi*	17	622.5	1.9	χ^2^ = 0.7318 (*df* = 1, *p* = 0.392)
A5	pSL from *µi*	16	621.6	1.0	χ^2^ = 1.7375 (*df* = 1, *p* = 0.188)
A6	pSL from *πi*	15	620.6	0.0	χ^2^ = 1.4751 (*df* = 1, *p* = 0.225)
A7	Seg exp from *µi*	14	621.0	0.4	χ^2^ = 2.9855 (*df* = 1, *p* = 0.084)
A8*	pSP from *πi*	13	622.4	1.8	χ^2^ = 3.8517 (*df* = 1, *p* = 0.050)
A9	Prop pres from *πi*	12	626.2	5.6	χ^2^ = 6.2146 (*df* = 1, *p* = 0.013)
A10	pSP from *µi*	11	628.5	7.9	χ^2^ = 4.7247 (*df* = 1, *p* = 0.030)
	*Undaria SACFOR*				
S0	None	21	508.6	12.4	
S1	Seg exp from *µi*	20	505.9	9.7	χ^2^ = 0.0825 (*df* = 1, *p* = 0.774)
S2	Abund marina from *µi*	19	503.4	7.2	χ^2^ = 0.1655 (*df* = 1, *p* = 0.684)
S3	pSL from *µi*	18	501.1	4.9	χ^2^ = 0.3889 (*df* = 1, *p* = 0.533)
S4	Stability from *πi*	17	499.2	3.0	χ^2^ = 0.7475 (*df* = 1, *p* = 0.387)
S5	Seg exp from *πi*	16	497.4	1.2	χ^2^ = 0.8015 (*df* = 1, *p* = 0.371)
S6	pSL from *πi*	15	496.2	0.0	χ^2^ = 1.3112 (*df* = 1, *p* = 0.252)
S7	Dist marina from *µi*	14	496.5	0.3	χ^2^ = 2.8843 (*df* = 1, *p* = 0.089)
S8*	pLam from *πi*	13	497.6	1.4	χ^2^ = 3.4965 (*df* = 1, *p* = 0.062)
S9	Site exp from *πi*	12	500.6	4.4	χ^2^ = 5.4353 (*df* = 1, *p* = 0.020)
S10	pSP from *πi*	11	501.0	4.8	χ^2^ = 2.8707 (*df* = 1, *p* = 0.090)

A0 and S0 are full models containing all variables in the count (*µ*_*i*_) and zero (*π*_*i*_) portions of the model. Dropped term indicates the variable dropped at each stage of the backwards selection, with the likelihood ratio test comparing the new model to the preceding model. ∆AICc = difference to the lowest AICc value for each model. Selected optimal models are indicated by an asterisk. Dist marina = distance to nearest marina (km), Abund marina = abundance at nearest marina (n 0.25 m^−2^), Stability = substrate stability (%), Seg exp and Site exp = segment exposure and site exposure respectively (km), pSL and pSP = the percent cover of *Saccharina latissima* and *Saccorhiza polyschides* respectively (%), Prop pres = propagule pressure (km^2^)

**Table 3:** Optimal ZINB models for *Undaria* abundance (A8) and SACFOR (S8)

Model term	Coefficent value (β)	SE	z value	*p* value	% z value
*Undaria abundance (A8)*					
*µ*_*i*_					
Intercept (*α*)	2.719	0.503	5.41	< 0.001	18.24
pLam	− 2.618	0.424	− 6.18	< 0.001	20.84
pSP	− 0.960	0.425	− 2.26	0.024	7.62
Stability	0.026	0.006	4.06	< 0.001	13.69
Site exp	− 0.002	< 0.001	− 3.56	< 0.001	12.00
Prop pres	3.502	1.004	3.49	< 0.001	11.77
Log(*θ*)	0.994	0.212	4.70	< 0.001	15.85
*π*_*i*_					
Intercept (*α*)	0.621	2.383	0.26	0.794	1.70
pLam	5.243	1.696	3.09	0.002	20.26
Dist marina	0.497	0.145	3.43	< 0.001	22.49
Abund marina	− 0.357	0.094	− 3.82	< 0.001	25.05
Site exp	0.005	0.002	2.40	0.016	15.74
Prop pres	− 11.552	5.136	− 2.25	0.024	14.75
*Undaria SACFOR (A8)*					
*µ*_*i*_					
Intercept (*α*)	2.321	0.453	5.13	< 0.001	16.55
pLam	− 2.696	0.427	− 6.32	< 0.001	20.40
pSP	− 1.304	0.397	− 3.29	0.001	10.61
Stability	0.020	0.006	3.22	0.001	10.41
Site exp	− 0.002	0.000	− 3.90	< 0.001	12.60
Prop pres	3.390	0.931	3.64	< 0.001	11.76
Log(*θ*)	1.322	0.241	5.48	< 0.001	17.68
*π*_*i*_					
Intercept (*α*)	5.443	2.495	2.78	0.029	12.78
pSP	− 7.709	2.844	− 2.71	0.007	15.89
Dist marina	0.609	0.170	3.59	< 0.001	21.07
Abund marina	− 0.453	0.120	− 3.78	< 0.001	22.13
Site exp	0.007	0.003	2.40	0.016	14.06
Prop pres	− 13.437	5.599	− 2.40	0.016	14.07

Dist marina = distance to nearest marina (km), Abund marina = abundance at nearest marina (n 0.25 m^−2^), Stability = substrate stability (%), Site exp = site exposure (km), pLam and pSP = the percent cover of *Laminaria* spp. and *Saccorhiza polyschides* respectively (decimal  %), Prop pres = propagule pressure (km^2^), Log(θ) = link function between count (*µ*_*i*_) and zero (*π*_*i*_) portions of the model. % z value = absolute z value for a given term expressed as a percentage of total absolute z values for that portion of the model

Simple linear regression of observed values against fitted values from the optimal models indicated a significant model fit for both *Undaria* abundance (F(1, 138) = 586.7, *p* < 2.2e−16, Adj-R^2^ = 0.81) and SACFOR (F(1, 138) = 554.7, *p* < 2.2e−16, Adj-R^2^ = 0.80). Justification of model type (ZINB over a ZIP), was confirmed using likelihood ratio tests at the full (A0 and S0) and optimal (A8 and S8) model stages (χ^2^(A0) = 434.12, *df* = 1, *p* < 2.2e−16; χ^2^(A8) = 551.85, *df* = 1, *p* < 2.2e−16; χ^2^(S0) = 138.41, *df* = 1, *p* < 2.2e−16; χ^2^(S8) = 166.62, *df* = 1, *p* < 2.2e−16).

The relative importance of each term from the optimal models (% z value) suggests that distance to, and abundance at, the nearest marina had the most significant effect on the zero model for both *Undaria* abundance and SACFOR (Table 3). For the count model the percent cover of *Laminaria* spp. had the highest relative importance for both abundance and SACFOR variables (Table 3).

**Fig. 7:**
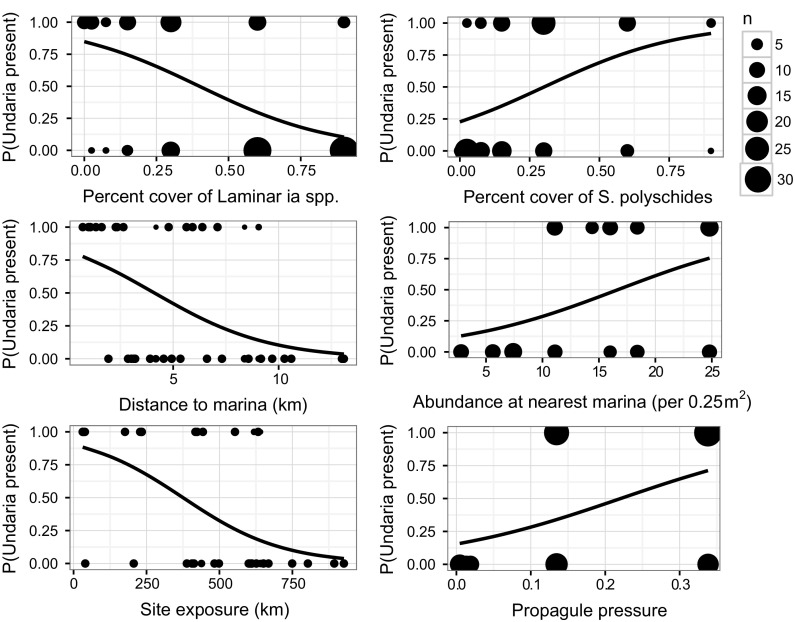
Relationship between key predictor variables selected in the zero portion of the optimal ZINB models (A8 and S8) and *Undaria* presence-absence. Size of points is equivalent to number of transects (n). Significance determined by binomial GLMs are indicated by plotted lines of fitted values for the probability of *Undaria* presence across the sampled range of the predictor

Scatterplots and binomial models of *Undaria* presence-absence data were used to further examine the relationship of each predictor variable selected in the optimal zero models (Fig. 7). Individually, all factors significantly affected the probability of *Undaria* presence, with the percent cover of *Laminaria* spp. (β = − 4.301, z = − 5.74, *p* = 9.78e−09), distance to nearest marina (β = − 0.366, z = − 5.06, *p* = 4.23e−07) and site exposure (β = − 0.006, z = − 5.81, *p* = 6.25e−09) all negatively related to *Undaria* presence; while the percent cover of *S. polyschides* (β = 4.042, z = 4.12, *p* = 3.75e−05), abundance at nearest marina (β = 0.138, z = 4.68, *p* = 2.90e−06) and propagule pressure (β = 7.708, z = 5.13, *p* = 2.87e−07) were all positively related to *Undaria* presence (Fig. 7).

**Fig. 8:**
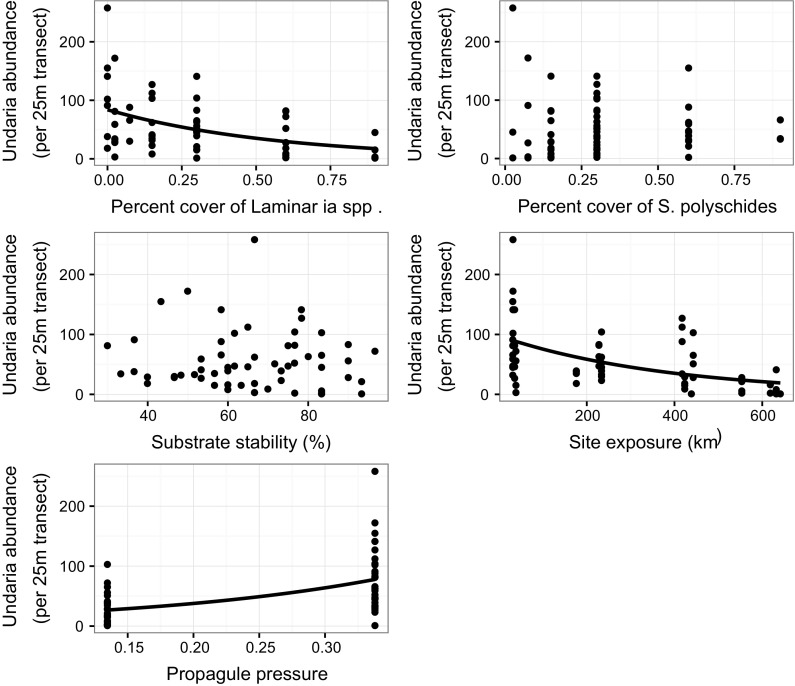
Relationship between key predictor variables selected in the count portion of the optimal ZINB model (A8) and *Undaria* abundance. Significance determined by negative binomial GLMs are indicated by plotted lines of fitted values for the abundance of *Undaria* across the sampled range of the predictor

**Fig. 9:**
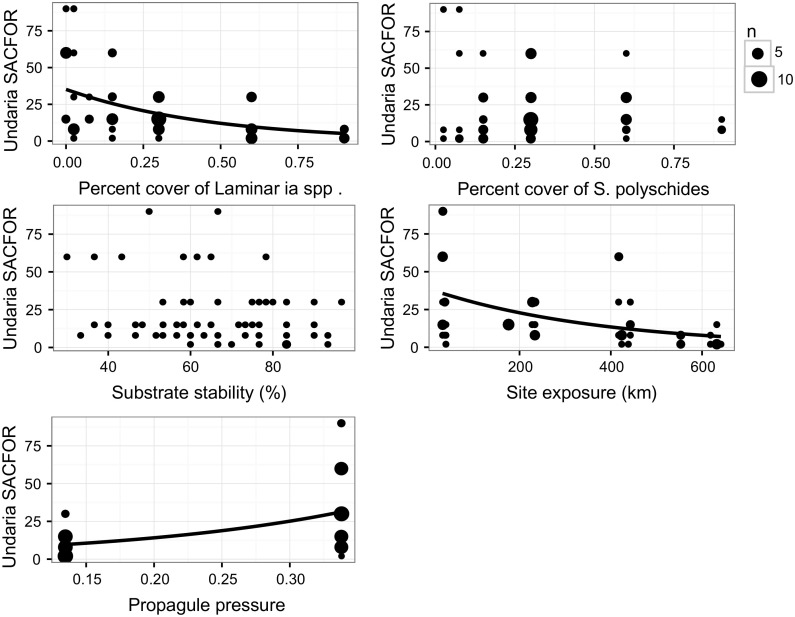
Relationship between key predictor variables selected in the count portion of the optimal ZINB model (S8) and *Undaria* SACFOR. Size of points is equivalent to number of transects (n). Significance determined by negative binomial GLMs are indicated by plotted lines of fitted values for *Undaria* SACFOR across the sampled range of the predictor

Negative binomial models of positive abundance data and individual variables selected in the count model of A8 indicated percent cover of *Laminaria* spp. (β = − 1.743, z = − 4.13, *p* = 3.66e−05) and site exposure (β = − 0.003, z = − 4.90, *p* = 9.37e−07) had a significant negative relationship with *Undaria* abundance; while propagule pressure (β = 5.247, z = 4.90, *p* = 9.68e−07) had a positive relationship (Fig. 8). Individually, substrate stability (β = − 0.004, z = − 0.59, *p* = 0.556) and the percent cover of *S. polyschides* (β = − 0.312, z = 0.55, *p* = 0.583) were not significantly related to *Undaria* abundance (Fig. 8). The same predictor variables were selected in the count portion of the optimal SACFOR model (S8), and negative binomial models indicated the same relationships as for the abundance model (Fig. 9) [(Percent cover of *Laminaria* spp. (β = − 2.137, z = − 5.37, *p* = 7.84e−08), site exposure (β = − 0.003, z = − 5.30, *p* = 1.19e−07), propagule pressure (β = 5.758, z = 5.66, *p* = 1.52e−08), substrate stability (β = − 0.014, z = − 1.92, *p* = 0.054), percent cover of *S. polyschides* (β = − 0.607, z = − 1.10, *p* = 0.272)].

## Discussion

The northeast Atlantic is a hotspot of *Undaria* invasion, yet a knowledge gap remains regarding the details of its invasion gateways and pathways (Fletcher and Farrell 1999; Floc’h et al. 1996; Minchin and Nunn 2014). Overall this study supports the hypothesis that artificial habitats facilitate a spillover and spread of *Undaria* to natural rocky reef (Cremades et al. 2006; Farrell and Fletcher 2006; Floc’h et al. 1996; James and Shears 2016; Russell et al. 2008). Although this had been suggested for *Undaria* in the northeast Atlantic (Cremades et al. 2006; Farrell and Fletcher 2006; Floc’h et al. 1996; Grulois et al. 2011), it had yet to be robustly examined across multiple locations. In the southwest UK it seems that marinas act as ‘strongholds’ for *Undaria* and in many cases the species is restricted to these habitats. Attributes of the marinas themselves, including their proximity to reef sites and the abundance and propagule pressure of *Undaria* they supported, had the strongest relationships with presence/absence patterns of *Undaria* within natural reef habitats. However, attributes of the recipient site, particularly the structure of the native macroalgal canopy and wave exposure, also strongly influenced the probability of *Undaria* occurrence. When *Undaria* was present, natural biotic and abiotic factors including the percent cover of *Laminaria* spp. and wave exposure had the largest impact on the abundance and cover of *Undaria*.

*Undaria* is now a dominant fouling species in marinas across the southwest UK. This is unsurprising given its ability to proliferate on artificial substrates (Cremades et al. 2006; Fletcher and Farrell 1999; Floc’h et al. 1996; Kaplanis et al. 2016; Russell et al. 2008; Veiga et al. 2014) and its prevalence in UK marinas (Fletcher and Farrell 1999; Heiser et al. 2014; Kraan 2016; NBN 2017). Indeed, *Undaria* is now more abundant than native kelp species at most marinas surveyed during the current study (Epstein pers obs.). This observation would support disturbance experiments which indicate that *Undaria* may out-compete native seaweeds in artificial habitats, including marinas within the UK (Curiel et al. 2001; Farrell and Fletcher 2006). There was, however, high variation in the abundance, biomass and percent cover of *Undaria* between marinas in this study. This is likely to be based on a variety of biotic and abiotic factors including competition, disturbance, temperature and light (Farrell and Fletcher 2006; Schiel and Thompson 2012).

The widespread development of marinas across the UK is creating large surface areas of artificial hard substrate, which is held at a constant shallow depth in sheltered conditions; ideal for *Undaria* growth (Farrell and Fletcher 2006; Kaplanis et al. 2016; Minchin and Nunn 2014; Veiga et al. 2014). Maximum areal extent of marinas within a given location was over 0.5 km^2^ (Plymouth Sound), with Torbay (0.29 km^2^) and Dartmouth (0.20 km^2^) also having considerable total surface areas of marinas. The high abundance and spatial coverage of *Undaria* on these substrates creates considerable propagule pressure; and therefore some spillover of *Undaria* to nearby natural habitats could be expected. This study shows, however, that in many cases *Undaria* is confined to marina habitats. *Undaria* was recorded on natural rocky reef in only 2 of the 5 locations (i.e. 40%), 17 of 35 sites (49%), and 60 of 140 transects (43%). This confinement to marina or harbour environments is not uncommon for the species and is similar to non-native populations from other locations including the USA (Kaplanis et al. 2016; Silva et al. 2002) and Portugal (Veiga et al. 2014). It has been suggested that *Undaria* may have a lower competitive ability in natural habitats, which may account for its confinement to artificial substrates in certain areas (Curiel et al. 2001; De Leij et al. 2017; Dellatorre et al. 2014; Edgar et al. 2004; Farrell and Fletcher 2006; Floc’h et al. 1996; Forrest and Taylor 2002; Valentine and Johnson 2003).

Where *Undaria* was present on natural substrates its abundance and percent cover was highly variable over relatively small spatial scales. In both Torbay and Plymouth Sound *Undaria* ranged from Superabundant/Abundant to Rare, with as few as 1 or 2 plants seen at some sites compared to well over 100 within a single transect at many others. As with findings from *Undaria* distribution studies in many other locations (Castric-Fey et al. 1993; Cremades et al. 2006; Martin and Bastida 2008; Russell et al. 2008), this suggests that when *Undaria* has colonised natural habitats there are a variety of factors which will influence its abundance and proliferation.

The two factors that had the strongest relationships with *Undaria* presence-absence at rocky reef sites were the distance to nearest marina and *Undaria* abundance at the nearest marina. For example, *Undaria* was not recorded at any sites > 9.1 km away from the nearest marina, or where the nearest marina had an abundance of < 11.1 individuals per 0.25 m^2^. These factors had a similar relationship to the abundance and cover of *Undaria* in natural habitats. Marina propagule pressure also had a significant relationship with *Undaria* presence-absence, abundance and percent cover. Indeed, *Undaria* was not recorded at any sites with propagule pressure under 0.13, and was more abundant and prominent within macroalgal canopies in locations with higher propagule pressure.

These patterns support the idea that the presence of *Undaria* on coastal reefs is heavily influenced by the build-up and eventual ‘spillover’ from artificial habitats. In many parts of its non-native range (New Zealand, Spain, France and the UK), the spillover from artificial to natural habitats has been suggested as an important mechanism in its invasion dynamics. This includes from harbours and marinas (Cremades et al. 2006; Farrell and Fletcher 2006; Floc’h et al. 1996; Russell et al. 2008), aquaculture sites (Cremades et al. 2006; James and Shears 2016) and algal mariculture sites (Floc’h et al. 1996). This study provides the first empirical evidence of spillover from artificial to natural habitats in the UK.

The spillover can, however, be a slow process. In the UK *Undaria* was present in a marina for 7 years before it was found on the adjacent rocky shoreline only 200 m away (Farrell and Fletcher 2006); while in a harbour of New Zealand *Undaria* was widespread in artificial habitats but, took 9 years to spread to natural substrates (Russell et al. 2008). The date of first record at a given marina is likely to give a poor estimate of true residence time, therefore this factor was not used in the statistical analysis of this study (see “Methods” section). Residence time is, however, likely to be a factor that influences the abundance and percent cover of *Undaria* at marinas, consequent propagule pressure at nearby reefs, and therefore its potential spillover. Within this survey the two locations with longest known introductions (Plymouth and Torbay) were the only two locations where *Undaria* was recorded on rocky-reef. The locations with shortest times since first record (Newlyn and Dartmouth, 3 and 5 years respectively) also had the lowest abundance, and one of the lowest percent covers at marinas. Over time, if the abundance and percent cover of *Undaria* increases at these sites, a spillover to reefs may become more likely. However, *Undaria* was absent along the rocky shoreline of Salcombe, despite the fact that it has persisted in the marina for at least 10 years, which may be due to other factors affecting overall propagule pressure, such as areal extent of marinas or the connectivity to nearby reef. Lag-time may be due to a slow build-up of propagule pressure in artificial habitats, eventually reaching a threshold which promotes spillover into natural habitats. In this study, the greater probability of occurrence, abundance and cover of *Undaria* at coastal sites in locations with higher marina propagule pressure lends support for this mechanism.

It should be noted that this survey specifically investigated marina populations, and a number of stratified rocky-reef sites around these marinas. There is potential for *Undaria* to be present on other artificial and natural substrates which were not included as part of this survey, which may therefore influence the distribution-abundance patterns of *Undaria* at the surveyed marina and reef sites. However, the survey design was considered appropriate to elucidate the link between marinas and the spread and distribution of *Undaria* at rocky-reef sites and was optimal given the constraints of the available time and resources. Further studies should investigate the influence of other artificial substrates on the spread of *Undaria*. Structures such as moorings, coastal defence, piers and bridges could act as important stepping-stones for further dispersal.

The structure of the native brown macroalgal canopy was strongly related to *Undaria* populations in natural reef habitats, as a lower coverage of *Laminaria* spp. was associated with a higher probability of occurrence and greater abundance and percent cover of *Undaria*. *Laminaria* spp. (i.e. *L. digitata*, *L. hyperborea* and *L. ochroleuca*) are the dominant canopy forming macroalgae along open rocky coastlines of the northeast Atlantic (Smale et al. 2013). They are large, long-lived perennial macroalgae with high competitive ability (Bartsch et al. 2008; Smale et al. 2013). In comparison, *Undaria* is considered to be opportunistic, with a fast growth rate, a short annual life-cycle and high investment in reproductive output (Choi et al. 2007; Saito 1975; Schiel and Thompson 2012). As such, it has been suggested that *Undaria* would be competitively inferior to *Laminaria* spp. in natural reef habitats of the UK (De Leij et al. 2017; Farrell and Fletcher 2006; Fletcher and Farrell 1999; Heiser et al. 2014; Minchin and Nunn 2014). In its native Japan and Korea, *Undaria* functions as a pioneer species in many environments, typical of early successional stages (Agatsuma et al. 1997; Kim et al. 2016), and this opportunistic strategy is seemingly mirrored in many parts of its non-native range. Disturbance to native canopies is often key to the recruitment and proliferation of non-native *Undaria* (Carnell and Keough 2014; De Leij et al. 2017; Thompson and Schiel 2012; Valentine and Johnson 2003), and distributional studies in France, New Zealand, Argentina and the UK have shown that *Undaria* occurs more commonly and at higher abundance when native macroalgal canopies have less cover (Castric-Fey et al. 1993; De Leij et al. 2017; Dellatorre et al. 2014; Heiser et al. 2014; Jiménez et al. 2015; Martin and Bastida 2008; South and Thomsen 2016). The results of this study support these findings across multiple sites and locations, with *Laminaria* spp. exerting a strong influence over *Undaria* presence-absence, abundance and percent cover. As the persistence of dense, intact *Laminaria* canopies may restrict the proliferation of *Undaria* in rocky reef habitats, preserving this biotic resistance by maintaining good environmental conditions could provide an additional management option to the direct control or exclusion of *Undaria*.

*Undaria* was, however, found in 16 transects where *Laminaria* spp. percent cover was recorded as Abundant (40–79%) or Superabundant (> 80%). In the majority of these transects *Undaria* was Rare or Occasional; however at three sites (Jennycliff, Fort Bovisand and Beacon Cove) *Undaria* was recorded as Common with an abundance of > 70 per 25 m transect. Although the native *Laminaria* canopy in the UK seems to have an inhibitive effect on *Undaria* (De Leij et al. 2017; Farrell and Fletcher 2006), these results indicate that in certain conditions they are able to co-exist at relatively high abundance and percent cover. These refugia among dense native canopies may allow *Undaria* to build up propagule pressure within natural substrates; removal or disturbance of the native canopy may therefore not be a proviso to *Undaria* presence or spread.

The relationship between *Undaria* and *S. polyschides* was less clear. In the ZINB model a higher coverage of *S. polyschides* was positively associated with occurrence of *Undaria*, but had a negative relationship with abundance and percent cover of *Undaria*. Further investigation showed a significant pattern of co-occurrence of the two species, but the negative relationship with abundance and cover of *Undaria* was less well defined. *Undaria* and *S. polyschides* are known to have a similar niche and life history (Castric-Fey et al. 1993; Norton and Burrows 1969; Yesson et al. 2015). Both are annual kelps with peak recruitment in late winter to early spring, maximal growth and biomass in late spring, and senescence through autumn (Castric-Fey et al. 1999a; Fletcher and Farrell 1999; Floc’h et al. 1991; Norton and Burrows 1969). They are both opportunistic when compared to *Laminaria* spp., with high growth rates and reproductive outputs, and are both found at highest abundance and cover in the low intertidal-shallow subtidal fringe (Castric-Fey et al. 1993, 1999b; Fletcher and Farrell 1999; Floc’h et al. 1991, 1996; Norton and Burrows 1969). The positive relationship recorded between *Undaria* occurrence and *S. polyschides* cover may be indicative of overlapping niches. However, due to their similarities, the presence of direct competition between these species has previously been suggested (Castric-Fey et al. 1993; Farrell and Fletcher 2006; Fletcher and Farrell 1999). This could be the cause of the negative relationship between *Undaria* abundance/cover and *S. polyschides* cover found within this study; perhaps with *Undaria* outcompeting *S. polyschides* under certain environmental conditions.

Wave exposure was also an important determinant of *Undaria* presence-absence, abundance and percent cover in natural habitats as *Undaria* was not recorded at sites with total wave fetch > 642 km, while abundance and cover was generally greater at more sheltered sites. Across its native and non-native range *Undaria* is generally found at highest abundance in sheltered to moderately-exposed open coasts or bays near the open sea (Floc’h et al. 1996; Russell et al. 2008; Saito 1975). Due to the thin fragile nature of its lamina *Undaria* is susceptible to wave action and is generally absent from highly exposed shores (Choi et al. 2007; Yesson et al. 2015). Periods of low water motion are also needed for high natural recruitment, with spore adhesion optimal at low water velocities (Pang and Shan 2008; Saito 1975). This study showed that on coastal sites in the southwest UK *Undaria* is highly influenced by local scale differences in exposure and may be limited or excluded from some areas due to the lack of suitable rocky substrates in sheltered settings.

A similar study carried out in northern New Zealand investigated the association between *Undaria* in mussel farms (the key habitat for *Undaria* colonisation in that region) and adjacent rocky-reef (James and Shears 2016). Similar to this study, *Undaria* was more commonly found on artificial substrates where it also reached significantly higher abundance compared to natural reef sites. At natural reef sites *Undaria* was found at only 8 sites (compared to 33 mussel farm sites), and was most strongly related to distance from shore, mussel farm size and mean abundance at farms; *Undaria* was also most abundant at reef sites which were lacking native macroalgal canopies. This aligns closely with the current study, as distance to and abundance at marinas, and native competitors, were major factors influencing *Undaria* presence-abundance patterns at reef sites. Both studies therefore suggest the potential spillover effects from artificial habitat sources to natural substrates, while also recognising the influence of natural biotic factors. One discrepancy between the studies is the influence of wave exposure, which was not identified as a significant factor influencing reef populations in northern New Zealand (James and Shears 2016). The influence of wave exposure is likely to be hard to quantify, with very local scale variations able to alter recruitment success (Pang and Shan 2008; Russell et al. 2008; Saito 1975). *Undaria* has also been recorded in wave exposed environments in southern New Zealand (Russell et al. 2008), but is generally found in sheltered environments in its native range and across the northeast Atlantic (Cremades et al. 2006; Peteiro et al. 2016; Saito 1975; Yesson et al. 2015). This may be due to local scale differences in wave dynamics, other related biotic factors, or different quantification or ranges of wave exposure.

The growth, recruitment and life-history of *Undaria* is known to be influenced by other environmental factors including light, salinity, nutrients and temperature (Floc’h et al. 1991; Gao et al. 2013; James and Shears 2016; Murphy et al. 2017; Saito 1975). Although these factors may have affected the abundance and distribution of *Undaria* in this study, its wide physiological niche means that their influence is likely to be small. Within its non-native range *Undaria* is known to occur in high abundance from the open coast to more estuarine environments with lower salinity, higher sediment and nutrient loading (Curiel et al. 2001; Floc’h et al. 1991; Russell et al. 2008). *Undaria* sporophytes are able to survive salinities down to 11 psu and light compensation point can occur as low as 17 − < 5 µmol m^−2^ s^−1^ (Saito 1975; Watanabe et al. 2014). *Undaria* is also viable over a wide range of light regimes, with light compensation point of sporophytes reached between 17 and < 5 μmol m^−2^ s^−1^ and the gametophyte requiring irradiances over just 3 μmol m^−2^ s^−1^ for growth and maturation (Campbell et al. 1999; Epstein and Smale 2017; Saito 1975; Watanabe et al. 2014), This study was also carried out over a latitudinal gradient of just 0.4˚ and within similar enclosed near-coast environments and, as such, did not encompass wide gradients in temperature, light and salinity. Studies conducted over larger spatial scales may identify temperature, light and salinity as important predictor variables for *Undaria* distribution patterns.

Although the patterns recorded in this study are highly likely to be associated with the physical and biological attributes of the environment, it should be noted that the findings are based on an observational survey, which is correlative in nature and cannot directly determine causation. Although challenging to implement, long-term monitoring and manipulative experiments would be needed to fully elucidate the influence of the biotic and abiotic factors on *Undaria* populations. Genetic methods may also be useful to identify the flow of individuals between habitats and locations. Such methods have been used to link *Undaria* populations from natural and artificial habitats in the Bay of St Malo (Brittany), for example (Grulois et al. 2011). Previous manipulative studies have also indicated the inhibitive effect of native perennial canopies on the abundance and distribution of *Undaria* in various regions (e.g. De Leij et al. 2017; Edgar et al. 2004; South and Thomsen 2016; Thompson and Schiel 2012; Valentine and Johnson 2003). However, further work, including long-term press-removals, disturbance experiments with long term monitoring and recruitment studies would yield a better understanding of the strength and direction of effects from the various biotic and abiotic factors identified in this survey. This is particularly needed in the northeast Atlantic where these types of studies are generally lacking.

Due to their conservation value and the variety of ecological goods and services they provide, managing the ecological impacts of NNS in natural habitats could be considered as a priority over artificial or anthropogenic environments (Kueffer and Daehler 2009). Where *Undaria* is confined to artificial habitats management may be deemed a low priority. However, the results of this study suggest that limiting the abundance and propagule pressure of *Undaria* in artificial habitats may restrict the likelihood of its spread and proliferation into surrounding natural rocky reef communities. Once present in natural habitats, the management or eradication of *Undaria* is highly challenging and often infeasible (Curiel et al. 2001; Hewitt et al. 2005; Thompson and Schiel 2012). Management could, therefore, be targeted to areas where *Undaria* is still confined to artificial habitats, but are considered at high risk of spillover to adjacent natural habitats.

Management within New Zealand between 1997 and 2009 targeted specific areas of artificial and natural substrates in order to limit the further spread of *Undaria* (Forrest and Hopkins 2013). Prolonged removal led to a large reduction in density on artificial port structures (1–5% of pre-managed density) and vessel infestation rates (31–56% of vessels infected in unmanaged ports, 0.06–1.3% infected in managed ports). Although this sustained regional-scale management effort was successful in limiting local populations, reintroduction and wider-scale spread still occurred, therefore making the cost and effort of management attempts hard to justify (Forrest and Hopkins 2013). In the current study, two of the survey locations (Plymouth Sound and Salcombe) are managed and protected under legal designations. In Plymouth Sound, *Undaria* is now a conspicuous component of native communities (Arnold et al. 2016; Heiser et al. 2014) and there is a pressing need to identify the level of ecological impact. Here, management actions aimed at reducing its abundance or spatial extent would likely be ineffective. In Salcombe, however, if *Undaria* is truly restricted to artificial habitats, management actions aimed at maintaining the biotic resistance of local native communities and limiting its abundance and propagule pressure within marinas could prove fruitful. This is likely to only be effective if accompanied by strict biosecurity (to avoid re-introduction) and long term commitments to management.

It is evident that NNS are now prevalent in the marine environment (Bax et al. 2003; Ruiz et al. 1997) and are often highly abundant in artificial habitats (Bulleri and Chapman 2010; Glasby et al. 2007; Ruiz et al. 2009). The potential for artificial structures to facilitate the spread of marine NNS both geographically and across different habitats has been highlighted for other non-native flora and fauna (Airoldi et al. 2015; Bax et al. 2003; Bulleri and Chapman 2010; Glasby et al. 2007; Ruiz et al. 2009). However, in many cases NNS remain constrained to these artificial habitats (Airoldi et al. 2015; Coutts and Forrest 2007; Dafforn et al. 2012). The exact mechanisms behind why some marine NNS remain constrained in their distribution, while others readily proliferate across multiple habitat types and wide spatial scales will be challenging to define. As shown for *Undaria*, spread of a NNS is likely to be strongly influenced by variability in propagule pressure and habitat suitability. Due to the interconnected nature of the marine environment, the risk of spillover to natural substrates over various temporal scales is inevitable, unless management or eradication of the NNS is implemented. Identifying high risk areas, natural boundaries and factors affecting the spread and abundance of NNS in natural habitats is key to future management prioritisation (Forrest et al. 2009). This study should allow better decisions to be made regarding the management of one of the most prolific invasive macroalgae in the UK.
